# Multi‐organ kinetic modeling for Na[^18^F]F pre‐clinical total‐body PET studies

**DOI:** 10.1002/mp.17499

**Published:** 2024-11-05

**Authors:** Jose Benitez‐Aurioles, Paul S. Clegg, Carlos J. Alcaide‐Corral, Catriona Wimberley, Adriana A. S. Tavares

**Affiliations:** ^1^ School of Physics & Astronomy University of Edinburgh Edinburgh UK; ^2^ University of Manchester, Division of Informatics Imaging and Data Science Manchester UK; ^3^ Edinburgh Imaging University of Edinburgh Edinburgh UK; ^4^ Centre for Clinical Brain Sciences University of Edinburgh Edinburgh UK; ^5^ University/BHF Centre for Cardiovascular Science University of Edinburgh Edinburgh UK

**Keywords:** calcification, kinetic modeling, Na[^18^F]F, perfusion, total‐body PET

## Abstract

**Background:**

Total‐body positron emission tomography (PET), already well‐established in the pre‐clinical setting, makes it possible to study multi‐parameters in biological systems as a whole, rather than focusing on single tissues analysis. Simultaneous kinetic analysis of multiple organs poses some daunting new challenges.

**Purpose:**

To explore quantifying the pharmacokinetics of Na[^18^F]F in multiple dissimilar murine organs simultaneously in vivo with total‐body PET imaging using different compartmental models for each organ and a shared cardiovascular system.

**Methods:**

Six mice underwent a 60‐min total‐body PET scan following intravenous bolus injection of Na[^18^F]F. Compartmental models were constructed for each organ (heart, lungs, liver, kidneys, and bone) using an image derived input function. Non‐linear least squares fitting of a model that connects the five organs to a shared cardiovascular system was used to analyze both the first 3 min of data and the full hour. Analysis was repeated 5000 times using different initial parameter values for each duration, permitting analysis of correlations between parameters.

**Results:**

The models give a good qualitative account of the activity curves irrespective of the duration of the data; however, the quality of the fits to 3 min of data (average $\chi_{\nu }^{2}$ is 2.72) was generally better. Comparison of perfusion values to literature values was possible for the liver and lungs with the former (liver, 0.540 ± 0.177 mL/ml/min) being well‐above expectations and the latter (lungs, 0.184 ± 0.413 mL/ml/min) in rough agreement. Correlations between microparameter values (especially affecting *k_2_*) caused very noticeable problems for data modeling from both the kidneys and the femur.

**Conclusion:**

The present study demonstrates an approach to performing kinetic modeling for multiple organs simultaneously with Na[^18^F]F. The observed correlations between microparameter values remain a challenge. Nonetheless, many microparameters can be estimated reliably with a quantitative analysis of perfusion being possible for some organs.

## 1 INTRODUCTION

Total‐body positron emission tomography (PET) clinical systems with extended fields of view have been proposed as a solution to the current physical limitations of PET systems that have a limited field of view. Indeed, due to their geometry, current PET scanners have low sensitivity, which results in the low spatial and temporal resolution of their images^1^ The development of extended field‐of‐view scanners like the uExplorer (United Imaging),^2^ the PennPET (University of Pennsylvania),^3^ and the visionQuadra (Siemens)^4^ can improve the sensitivity of current scanners up to 40‐fold and their signal‐to‐noise ratio six‐fold.^5^ These enhancements allow for lower doses, shorter scans, and higher resolutions in both static and dynamic studies, hence expanding the clinical and research capabilities of PET technology. However, beyond these technical improvements, total‐body PET creates the opportunity to study biological systems as a whole, instead of focusing on single tissues. Indeed, multi‐organ processes and diseases are increasingly investigated, such as the gut‐brain axis^6^ or the pathological connections between the heart and liver^7^ as well as the heart and brain.^8^ Such investigations would be well‐supported by PET data analysis approaches, which highlight organs as a coupled system. Total‐body PET has been widely available in the preclinical setting for a few years, namely total‐body PET imaging of mice. The use of pre‐clinical scanners, which, if not boasting the same sensitivity advantages as the new human equivalents, allow researchers to explore systems‐level interactions in the body and, importantly, validate those exploratory systems biology studies with invasive tissue sampling that is not possible in humans. Therefore, preclinical total‐body PET imaging could be an important precursor to clinical total‐body PET by enabling testing and validation of new paradigms for quantification of imaging data.

Na[^18^F]F is a radiotracer, which has traditionally been used for its deposition in bone and bone lesions, particularly in oncological studies to identify and assess bone metastases.^9^, ^10^, ^11^ However, new studies have explored its use to detect and measure micro‐calcification around the body, particularly in the heart and vasculature, as a biomarker for heart diseases such as coronary artery disease or aortic stenosis.^12^, ^13^, ^14^ Recent quantification approaches gain predictive accuracy by using other imaging modalities to delineate the coronary vasculature before quantifying the Na[^18^F]F activity within it.^15^ Furthermore, micro‐calcification detected with Na[^18^F]F has also been detected in the aorta^16^ and femoral vessels,^17^ thus illustrating multi‐site/multi tissue uptake in distant regions of the body. Vascular networks irrigate all organs and tissues in the body, thus other applications of Na[^18^F]F at a systems level are easily envisioned. Therefore, there is a need to better understand and quantify Na[^18^F]F multi‐tissue dynamics and kinetics. Importantly, research with Na[^18^F]F has exclusively studied its ability to quantify calcification and micro‐calcification with simplified outcome measures. Very recently, in the context of being able to recognize when fluoride ions are detaching from complex tracer molecules, a dynamic PET study of rats has been carried out using the tracer Na[^18^F]F. Here the data was analyzed using pharmacokinetic modeling of multiple organs independently, but perfusion was not quantified.^18^ Hence, the capability of Na[^18^F]F as a proxy to quantify perfusion, or blood flow, for a broad range of organs are yet to be explored. Indeed, the possibility to quantify both micro‐calcification and perfusion across multiple organs and tissues in the whole‐body could strengthen the diagnostic value of PET images in a multitude of contexts.

Compartmental modeling of dynamic PET data allows us to extract pharmacokinetic parameters that are analogous to perfusion.^19^ One core element of this type of modeling is the need for dynamic information about the radiotracer concentration in arterial blood, preferably from a vessel near the studied tissue, called the arterial input function (AIF). However, accurate and frequent sampling of arterial blood during the PET scan through cannulation can be uncomfortable, especially for frail patients.^20^ Some total‐body PET kinetic modeling studies have been carried out, mostly with graphical analysis methods (Patlak plots) rather than compartmental modeling.^21^, ^22^ More recently, compartmental modeling has been given increasing attention for the tracer 2‐[^18^F]‐fluoro‐2‐deoxy‐d‐glucose (^18^F‐FDG)^23^, ^24^, ^25^ including consideration of model selection.^26^ The latter work highlights the importance of including the time delay. Giving an account of the time evolution of the activity for multiple organs simultaneously for other tracers is an essential step to harnessing the power of total‐body PET.

This study aims to investigate and quantify the pharmacokinetics of Na[^18^F]F in multiple dissimilar murine organs simultaneously in vivo with total‐body PET imaging using different compartmental models for each organ and to compare performance for different acquisition lengths. Here, we report the development of a model that connects the five organs under investigation to a shared cardiovascular system, which we fit to two sets of data, one that simulates a short, 3 min scan, and one simulating a longer, 60 min scan. The organs have been chosen to span a range of different pharmacokinetic models. Via the shorter analysis, we examine whether dynamic information, including perfusion, could be estimated using a shorter scan protocol. The overarching aim of this study is to help formal model selection for multi‐organ kinetic analysis of total‐body PET data^27^ using the example of the tracer, Na[^18^F]F. The uptake rate of each organ (*K_1_*) is considered in detail. In spite of the importance of the mouse as a pre‐clinical model^28^ the current pre‐clinical approach would require some detailed tuning to adapt it to the context of clinical total‐body PET.

## 2 MATERIALS AND METHODS

### 2.1 Na[^18^F]F radiosynthesis

^18^F‐fluoride was produced in a cyclotron and harvested as Na[^18^F]F formulated in saline (0.9% w/v) using a FASTlab synthesizer (GE Healthcare) and commercially available cassettes.

### 2.2 Animals

For this study, six naive adult male mice aged 13 weeks (38 ± 2 g, mean ± SD) were housed and maintained at the Edinburgh Preclinical Imaging Facility, University of Edinburgh, UK, with food and water available ad libitum and a standard cycle of 12 h light:12 h dark conditions. The experiments reported here were performed in accordance with the ARRIVE guidelines^29^ approved by the local University of Edinburgh animal ethics committee, and authorized by the Home Office under the Animals (Scientific Procedures) Act 1986.

### 2.3 Na[^18^F]F PET Studies

The mice were transferred to the preclinical PET/CT scanner (nanoPET/CT, Mediso, Hungary). General anesthesia was maintained throughout the duration of the PET/CT study (0.5/0.5 L/min of oxygen/nitrous oxide and 2.0% isoflurane); vital signs, including temperature and respiration rate, were monitored during the experiments. Mice received an intravenous tail‐vein bolus injection of Na[^18^F]F (12 ± 8 MBq, mean ± SD, *n* = 6). The injected dose was chosen to ensure a good signal to noise ratio and quantitative accuracy for small regions while remaining consistent with the radiotracer principle. The animals immediately underwent a 60‐min total‐body emission scan, which is sufficiently long to capture the full kinetic behavior.^30^ A CT scan (semi‐circular full trajectory, maximum field of view, 360 projections, 50kVp, 300 ms, and 1:4 binning) was conducted at the end of each PET scan. Attenuation correction was conducted, and the PET and CT images were aligned for analysis. All PET images were reconstructed into 18 × 10, 2 × 30, 1 × 60, 2 × 120, 10 × 300 s frames using Mediso's iterative Tera‐tomo 3D reconstruction algorithm and the following settings: four iterations, six subsets, full detector model, low regularization, spike filter on, voxel size 0.4 mm, and 400–600 keV energy window. PET data were corrected for random coincidences, scatter and attenuation. Volumes of interest (VOIs) were drawn (using PMOD 3.117 software, PMOD Technologies, Switzerland) on reconstructed scans around organs of interest (heart, lungs, liver, and bone) using the CT data with the exception of the kidneys and the vena cava, where the PET data was used. Using the framing described above, time‐activity curves (TACs) were determined for each organ.

### 2.4 Compartmental model

**FIGURE 1:**
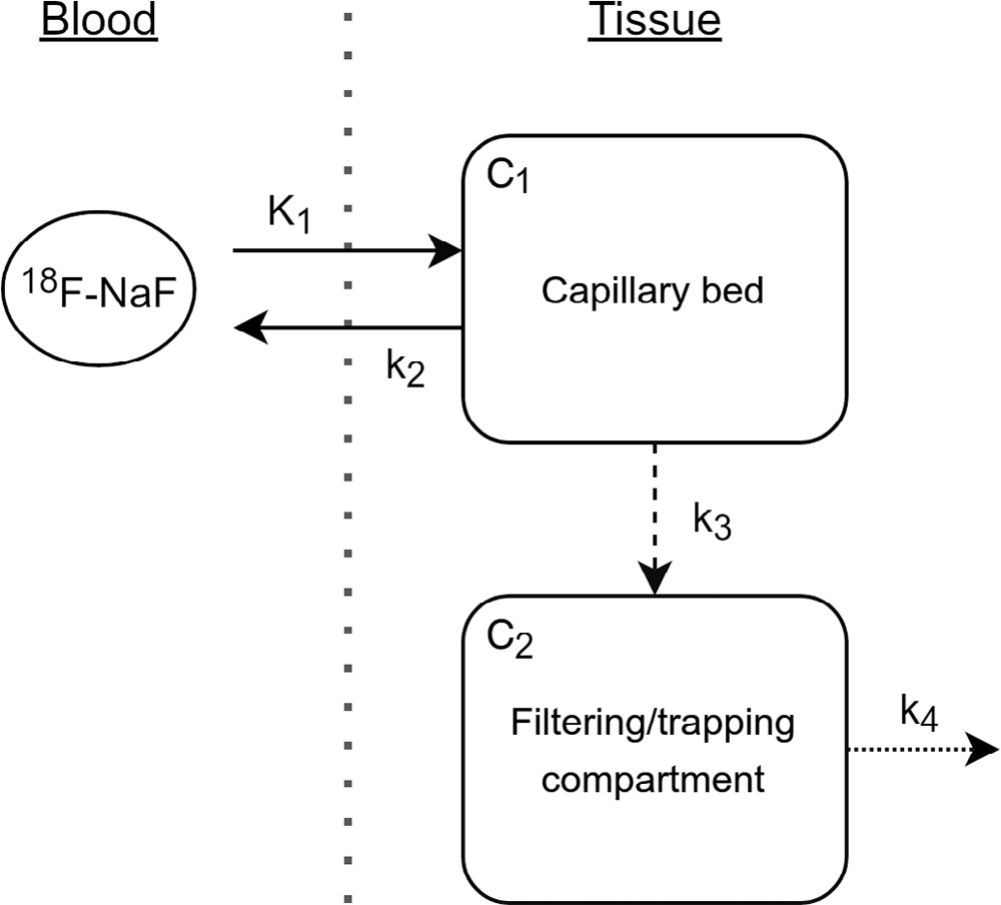
Compartmental model used for analysis. For the heart, lungs, liver and femur, k_4 _= 0 min^−1^ and for the heart, lungs, and liver k_3 _= 0 min^−1.^

Three different compartmental configurations were used, depending on the tissue (Figure 1). For the heart, liver, and lungs, a one‐compartment model, with a tracer concentration *C_1_*, was used to simulate the nutritive flow of blood into these organs. *K_1_* represents the uptake rate of radiotracer from blood in the capillary bed to tissue parenchyma, while *k_2_* denotes the transport of tracer back into the cardiovascular circulation. In the case of these three organs, a volume of distribution relationship was used between these two parameters, so that *V_d_* = *K_1_*/*k_2_* is approximated as the percentage of water in the tissue.^31^ Using wet weight data from,^32^ the volume of distribution was set as 0.665 mL/cm^3^ for the heart, 0.763 mL/cm^3^ for the lungs, and 0.751 mL/cm^3^ for the liver. For the femur, an additional compartment of concentration *C_2_*, was added, which irreversibly traps the radiotracer through a trapping rate *k_3_*, simulating active and irreversible incorporation of Na[^18^F]F into bone hydroxyapatite crystals. For the kidneys, the radiotracer was in addition removed from the second compartment through a clearance to the bladder rate *k_4_*. For the latter two tissues, no restrictions on the relationship between *K_1_* and *k_2_* is imposed because it is anticipated that Na[^18^F]F versus ^15^O‐water will have different tissue kinetics. The choice of compartmental model for each tissue was informed both on the expected pharmacokinetic behavior of Na[^18^F]F and time‐activity curves observable kinetics.

### 2.5 Arterial input function

The five tissues’ compartments are connected to one common cardiovascular blood system, which has an activity curve *C_a_* called the AIF. An image‐derived input function (IDIF) *C_IDIF_* was recovered for each mouse by using the PET scan activity data of the vena cava, a well‐established approach.^33^, ^34^ Here, the activity of the blood can be clearly isolated.^30^ It is justifiable to use venous blood from the vena cava as a proxy for the AIF both because the first‐pass extraction fraction is sufficiently low and because the venous to arterial differences seen in humans are less of a concern for mice. A mouse heart beats around 350 times per minute, meaning that venous and arterial compartments are well mixed, as shown in Figure 1b of Ref.^30^

Three corrections were introduced to the model to account for the differences between the IDIF from the vena cava and the true AIF. First, to account for partial volume effects,^35^ the magnitude of the AIF is divided by a correction factor γ. Second, to consider the deformation of the activity curve as the radiotracer is diffused throughout the bloodstream, a convolution filter is applied to the AIF as $\frac{1}{\tau }e^{-t\;/\;\tau }\;⊗\;C_{a}\left(t\right)$ where τ is the dispersion coefficient,^36^ which we have chosen to be equal in all tissues. While this is unlikely to be a perfect formulation, it is not feasible to estimate a separate dispersion coefficient for each organ without destabilizing other parameters. The approximation of uniform dispersion coefficients is likely to be reasonable for most tissues. For the liver, it can be assumed that the blood supply from the portal vein will be more highly dispersed making this approximation less accurate. To account for the difference in times at which the initial peak of radiotracer reaches each organ, separate delay parameters are considered for the heart, lungs, liver, kidneys, and femurs, labeled *Δt_h_*, *Δt_l_*, *Δt_i_*, *Δt_k_*, and *Δt_f_*.

Additionally, five blood volumes *v_bh_*, *v_bl_*, *v_bi_*, *v_bk_*, and *v_bf_*, defined as the fractions of signal that are measured in the VOIs of the heart, lungs, liver, kidneys, and femurs, respectively, by nearby vessels and vascularization, are also included in the models.

### 2.6 Noise model

A noise model related to counting statistics^37^ was used for the activity curves collected, of the form $∼N\left(t\right)\cdot Sc\cdot \sqrt{\frac{e^{\lambda t}\cdot C_{T}}{\Delta t}}$ where N(t) is the standard normal distribution, $C_{T}$ is the activity signal of the VOI, $e^{\lambda t}$ is a factor that account for the decay correction performed to the data, where *λ* is the decay constant of ^18^F, and Δt is the scan length of each dynamic time point. Sc is a scaling factor to account for scanning conditions, size of VOI and sensitivity, and is chosen qualitatively to match the observed noise in a particular organ.

The noise in the measurements of the output tissue signals is incorporated in the minimization algorithm we will outline in the next section. However, this does not apply to the input vena cava signal, which, due to being image derived, is especially noisy. To smooth out the input function curves for all mice, the PET signal is fitted by a model function, a sum of different exponentials,^38^ giving us an analytical expression for the IDIF.

### 2.7 Parameter estimation

To fit our compartmental model to the data, a Levenberg‐Marquardt least‐squares minimization is used,^39^ with the corrected IDIF as input. As outlined in the last section, the input function was fitted to an exponential model, and because of this all calculations necessary to correct the AIF and solve the compartmental model can be done analytically, giving us a final exponential form to fit to the data. This permits shorter computation times and the reduction of time‐discrete calculation errors associated with quantitative approximations. This is particularly relevant to calculations that involve the IDIF in a bolus injection context, which exhibits very fast changes for the first few minutes after the tracer enters the blood flow.

The compartmental model has 23 parameters in total to fit five organ TACs simultaneously with the IDIF as input, so to fully explore parameter space the fitting algorithm is repeated 5000 times, with initial parameters picked through lattice hypercube sampling.^40^ This has similarities to searching for equivalent fits to a single set of data.^41^ From the set of 5000 results, kernel density estimation was performed so as to identify the highest probability parameter result of the model for the observed data.

### 2.8 Performance assessment and statistical analysis

To assess the performance of the model for each set of data, we considered the reduced chi‐square $\chi_{\nu }^{2}$ of the minimizer result, defined as:

$$\;\chi_{v}^{2}=\frac{1}{v}\;\sum_{i}^{N}{\left(O_{i}-C_{i}\right)}^{2}$$

where ν is the degrees of freedom of the model (number of observations *N* minus the number of free parameters in the model), $O_{i}$ are the observations of the model, and $C_{i}$ are the calculated predictions of the fit. Error‐bars for each parameter are estimated by the amount the parameter varies when the $\chi_{\nu }^{2}$ of the result changes by 1. Pearson correlation coefficients were derived from the set of results close by a change in $\chi_{\nu }^{2}$ of 1 to our fit. The significance of the Pearson correlation coefficients (*r*) over the six mice was tested using the Wilcoxon signed‐rank test at the *p* = 0.05 level.

## 3 RESULTS

### 3.1 Comparison between model and data

**FIGURE 2:**
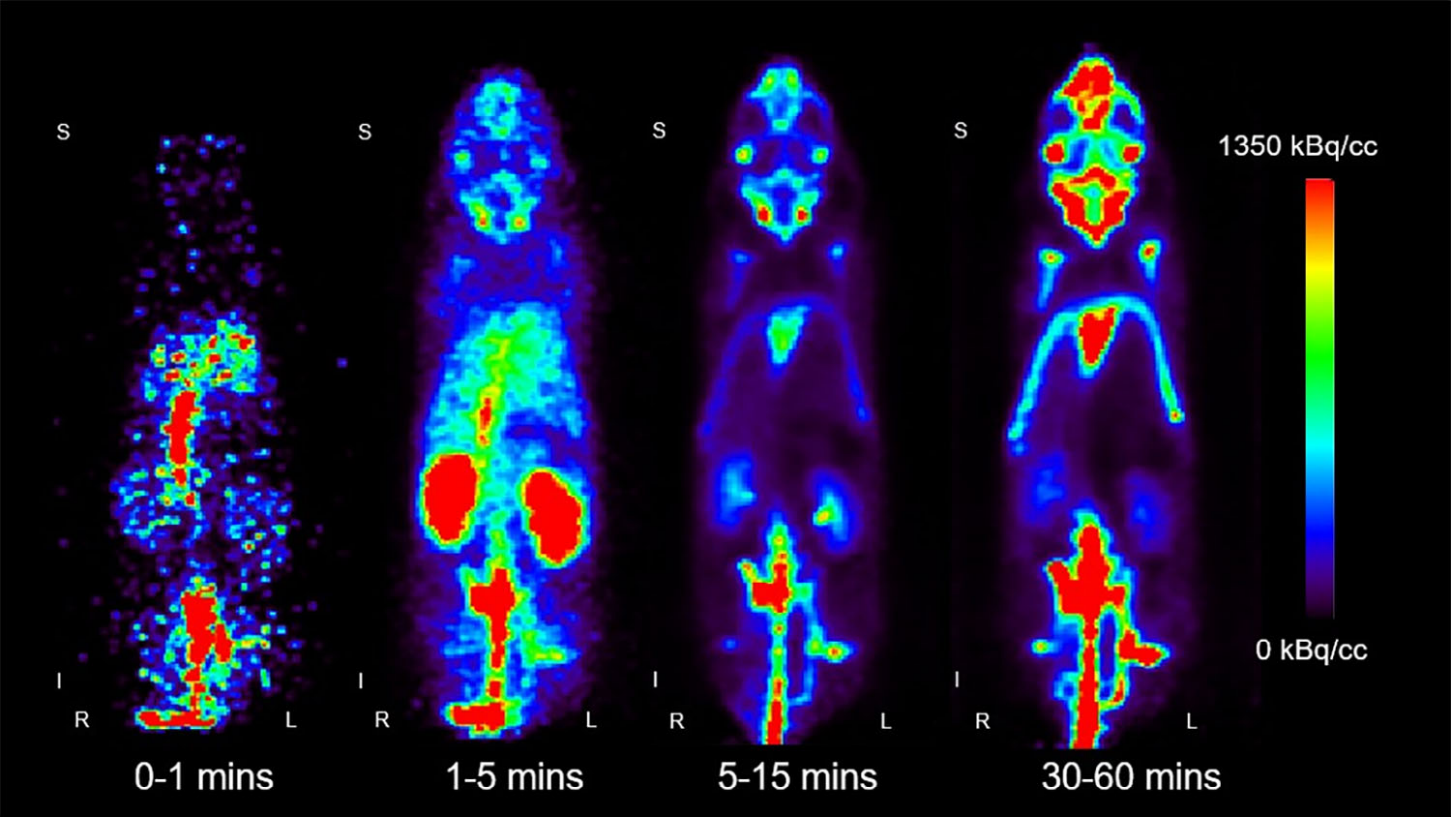
Coronal view of one representative Na[^18^F]F PET scan for four different time windows. R = right, L = left, S = superior, I = inferior. Qualitative differences in the kinetics of, for example, the lungs, kidneys, and bones are immediately obvious.

**FIGURE 3:**
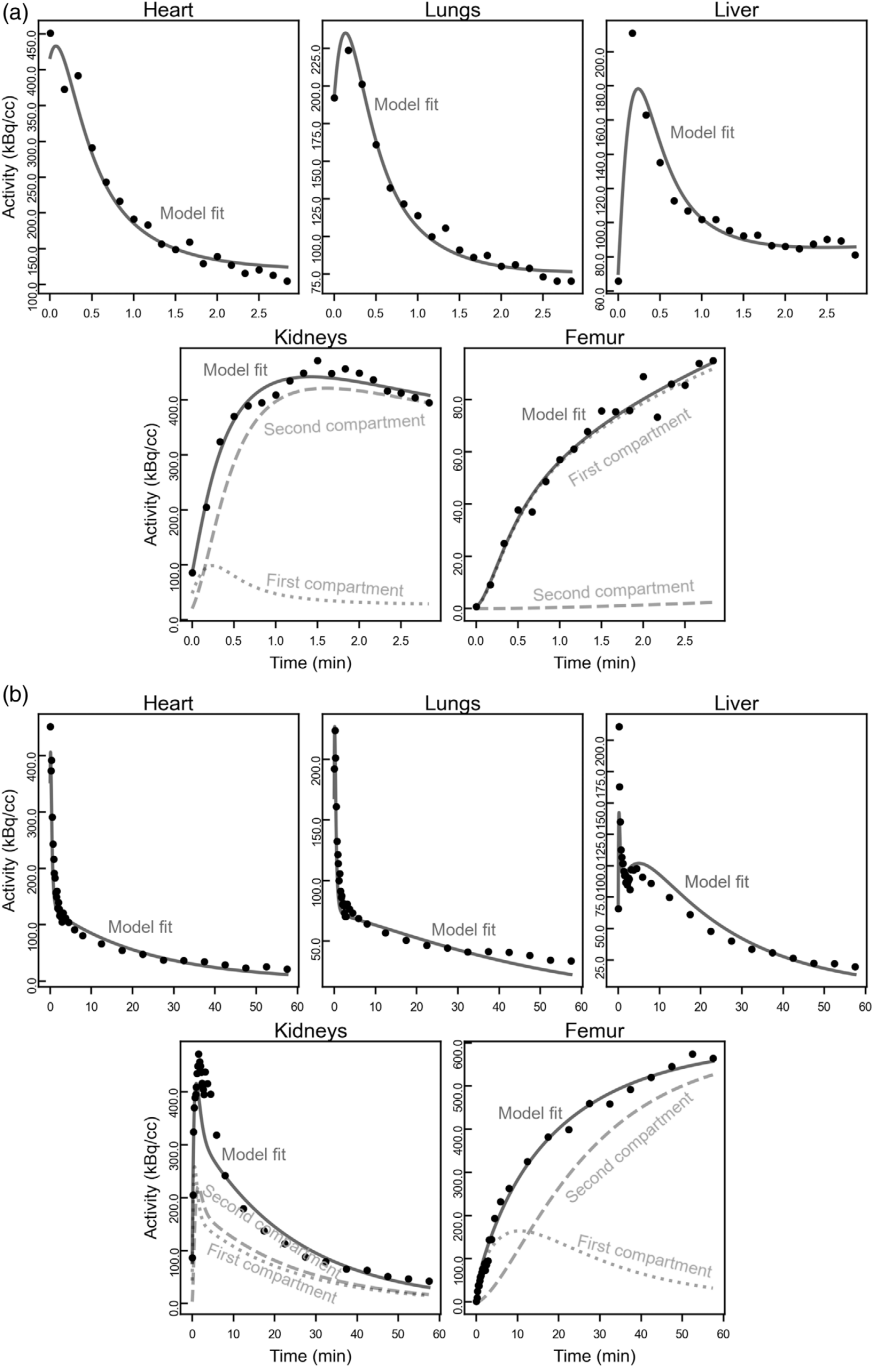
Compartment model fit to the PET activity curve data of the heart, lungs, liver, kidneys, and femurs of mouse 1, fitted to the first 3 min of the curve (a) and the full hour of data (b).

Representative Na[^18^F]F images of a mouse for different time windows are shown in Figure 2. Initially, intense radioactivity is seen for the heart and lungs. Next, the kidneys become strongly visible, and finally, the skeleton system becomes the most intense. Example activity curves with the associated model fits are presented in Figure 3a and 3b for the first 3 min and the full data, respectively. The resulting parameters are given in Table 1.

**TABLE 1:** Parameter estimations for each mouse, as estimated by the minimizer iterations.

	γ (dimensionless)		τ (min)		Δt_h_ (min)		Δt_l_ (min)		Δt_i_ (min)		Δt_k_ (min)		Δt_f_ (min)	
Mouse	Short scale	Long scale	Short scale	Long scale	Short scale	Long scale	Short scale	Long scale	Short scale	Long scale	Short scale	Long scale	Short scale	Long scale
16A0818A	0.65 ± 0.23	0.66 ± 0.12	0.18 ± 0.10	0.12 ± 0.19	0.21 ± 0.17	0.14 ± 0.14	0.04 ± 0.31	0.04 ± 0.06	0.13 ± 0.10	0.08 ± 0.10	0.12 ± 0.54	0.09 ± 0.05	0.01 ± 0.11	−0.22 ± 0.15
16A0818B	0.98 ± 0.11	0.91 ± 0.03	0.14 ± 0.04	0.06 ± 0.08	0.27 ± 0.04	0.26 ± 0.07	0.19 ± 0.15	0.20 ± 0.09	0.27 ± 0.07	0.22 ± 0.09	0.28 ± 0.09	0.22 ± 0.07	0.15 ± 0.06	0.07 ± 0.24
16A0823	0.84 ± 0.34	1.40 ± 0.01	0.11 ± 0.09	0.10 ± 0.03	0.46 ± 0.44	0.38 ± 0.02	0.38 ± 0.16	0.34 ± 0.04	0.44 ± 0.04	0.47 ± 0.00	0.48 ± 0.34	0.39 ± 0.03	0.36 ± 0.51	0.36 ± 0.22
17A1012C	0.82 ± 0.11	0.60 ± 0.01	0.24 ± 0.14	0.25 ± 0.07	0.35 ± 0.25	0.38 ± 0.03	0.32 ± 0.14	0.41 ± 0.01	0.41 ± 0.05	0.33 ± 0.02	0.35 ± 0.19	0.29 ± 0.06	0.35 ± 0.06	035 ± 0.06
17A1101A	0.68 ± 0.04	0.47 ± 0.00	0.21 ± 0.10	0.50 ± 0.01	0.38 ± 0.03	0.46 ± 0.00	0.33 ± 0.10	0.53 ± 0.00	0.40 ± 0.03	0.44 ± 0.00	0.39 ± 0.05	0.40 ± 0.04	0.26 ± 0.14	0.38 ± 0.25
17A1101B	0.62 ± 0.16	0.36 ± 0.01	0.36 ± 0.12	0.49 ± 0.02	0.40 ± 0.02	0.49 ± 0.01	0.35 ± 0.14	0.26 ± 0.04	0.40 ± 0.01	0.43 ± 0.01	0.41 ± 0.01	0.37 ± 0.08	0.28 ± 0.04	0.32 ± 0.02

	K_1h_ (ml/ml/min)		K_1l_ (ml/ml/min)		K_1i_ (ml/ml/min)		K_1k_ (ml/ml/min)		K_1f_ (ml/ml/min)	
Mouse	Short scale	Long scale	Short scale	Long scale	Short scale	Long scale	Short scale	Long scale	Short scale	Long scale
16A0818A	3.17 ± 0.82	14.52 ± 0.51^a^	0.04 ± 1.92	0.02 ± 0.01	0.13 ± 0.29	0.21 ± 0.16	2.55 ± 0.79	1.66 ± 0.33	0.17 ± 0.05	0.23 ± 0.09
16A0818B	14.98 ± 1.45^a^	0.54 ± 0.35	0.04 ± 0.03	0.02 ± 0.00	0.12 ± 0.36	0.22 ± 0.04	2.29 ± 0.32	2.58 ± 0.05	0.11 ± 0.02	0.18 ± 0.05
16A0823	14.05 ± 0.52^a^	5.72 ± 1.16	0.02 ± 0.14	6.00 ± 0.02^a^	0.12 ± 0.30	0.42 ± 0.15	2.26 ± 0.93	2.70 ± 0.20	0.38 ± 0.18	0.39 ± 0.05
17A1012C	4.98 ± 3.24	13.03 ± 0.68^a^	5.60 ± 3.14^a^	0.03 ± 0.00	1.68 ± 0.35	1.24 ± 0.27	2.52 ± 0.88	1.70 ± 0.35	0.90 ± 0.40	0.35 ± 0.09
17A1101A	3.87 ± 0.78	13.83 ± 0.95^a^	0.69 ± 0.46	0.20 ± 0.01	0.99 ± 0.31	0.86 ± 0.01	2.56 ± 0.34	1.95 ± 0.26	0.25 ± 0.04	0.23 ± 0.13
17A1101B	6.22 ± 0.99	15.00 ± 0.04^a^	0.13 ± 0.58	0.02 ± 0.00	0.20 ± 0.78	0.00 ± 0.00	4.01 ± 0.36	2.66 ± 0.20	0.34 ± 0.02	0.18 ± 0.01

	k_2k_ (/min)		k_3k_ (/min)		k_4k_ (/min)		k_2f_ (/min)		k_3f_ (/min)		k_if_ (ml/ml/min)	
Mouse	Short scale	Long scale	Short scale	Long scale	Short scale	Long scale	Short scale	Long scale	Short scale	Long scale	Short scale	Long scale
16A0818A	4.37 ± 0.71	0.00 ± 0.46	7.12 ± 1.48	1.68 ± 0.38	0.60 ± 0.26	1.51 ± 0.22	0.08 ± 0.05	0.06 ± 0.39	0.01 ± 0.63	0.09 ± 0.21	0.03 ± 0.17	0.15 ± 0.02
16A0818B	1.17 ± 0.14	2.27 ± 0.33	0.08 ± 0.04	4.20 ± 0.27	0.00 ± 0.15	0.88 ± 0.01	0.00 ± 0.13	0.01 ± 0.11	3.00 ± 0.00^a^	0.03 ± 0.26	0.11 ± 0.05	0.15 ± 0.02
16A0823	1.48 ± 0.94	0.00 ± 0.16	0.10 ± 0.71	2.41 ± 0.21	0.00 ± 0.57	2.42 ± 0.10	3.00 ± 0.98^a^	0.09 ± 0.02	3.00 ± 0.92^a^	0.06 ± 0.01	0.019 ± 0.06	0.15 ± 0.01
17A1012C	2.00 ± 1.53	0.00 ± 0.24	3.27 ± 4.90	1.51 ± 0.49	0.14 ± 0.56	1.39 ± 0.27	2.73 ± 1.49^a^	0.22 ± 0.17	1.02 ± 0.48	0.14 ± 0.04	0.25 ± 0.12	0.14 ± 0.02
17A1101A	0.09 ± 0.14	0.03 ± 0.29	1.38 ± 0.51	1.90 ± 0.53	0.91 ± 0.20	1.53 ± 0.62	0.63 ± 0.36	0.09 ± 0.19	2.72 ± 0.25	0.04 ± 0.04	0.20 ± 0.02	0.07 ± 0.02
17A1101B	5.44 ± 1.05	5.20 ± 0.25	1.23 ± 0.26	2.68 ± 0.22	0.00 ± 0.00	0.69 ± 0.09	2.96 ± 0.30	0.72 ± 0.11	1.66 ± 0.52	0.46 ± 0.07	0.12 ± 0.07	0.07 ± 0.00

	v_bh_ (ml/ml)		v_bl_ (ml/ml)		v_bi_ (ml/ml)		v_bk_ (ml/ml)		v_bf_ (ml/ml)	
Mouse	Short scale	Long scale	Short scale	Long scale	Short scale	Long scale	Short scale	Long scale	Short scale	Long scale
16A0818A	0.86 ± 0.34	0.39 ± 0.29	0.36 ± 0.13	0.25 ± 0.18	0.49 ± 0.29	0.43 ± 0.07	0.05 ± 0.08	0.09 ± 0.21	0.00 ± 0.01	0.00 ± 0.03
16A0818B	1.00 ± 0.26^a^	1.00 ± 0.08^a^	0.38 ± 0.11	0.29 ± 0.07	0.54 ± 0.03	0.48 ± 0.02	0.08 ± 0.22	0.18 ± 0.11	0.02 ± 0.01	−0.00 ± 0.01
16A0823	1.00 ± 0.74^a^	1.00 ± 0.26^a^	0.48 ± 0.20	0.78 ± 0.07	0.53 ± 0.23	0.00 ± 0.00	0.01 ± 0.39	0.13 ± 0.08	−0.03 ± 0.08	−0.01 ± 0.06
17A1012C	1.00 ± 0.08^a^	0.18 ± 0.06	0.15 ± 0.44	0.00 ± 0.00	0.00 ± 0.53	0.43 ± 0.00	0.04 ± 0.04	0.06 ± 0.09	0.02 ± 0.06	0.03 ± 0.06
17A1101A	1.00 ± 0.17^a^	0.64 ± 0.37	0.23 ± 0.16	0.00 ± 0.00	0.49 ± 0.13	0.59 ± 0.02	0.16 ± 0.13	0.24 ± 0.14	0.02 ± 0.03	−0.00 ± 0.01
17A1101B	1.00 ± 0.52^a^	0.01 ± 0.01	0.32 ± 0.32	0.35 ± 0.01	0.58 ± 0.03	0.32 ± 0.01	0.03 ± 0.03	0.14 ± 0.15	−0.01 ± 0.02	−0.00 ± 0.00

^a^ The minimiser diverged to the upper limit of the specific parameter.

The corresponding $\chi_{\nu }^{2}$ values are presented in Table 2 gathered for each mouse and in Table 3 gathered for each organ. In each case, values are given for fits to the first 3 min of data and also for fits to the full hour of data. Table 2 makes obvious that there is some variability among the data from different mice, but the $\chi_{\nu }^{2}$ values are always lower for analysis of the early data alone. Table 3 indicates numerically that the lungs and the kidneys prove particularly challenging when modeling the full hour of data. Visually (Figure 3b), it is clear that the model fails to explain some of the late phase behavior of the organs (e.g., > 30 min). For tissues approximated by one tissue compartment, the model fails to fit both the peak and tail of the activity curves, while for the kidneys, the width of the initial activity peak is underestimated. The difficulties in predicting the entirety of the pharmacokinetic behavior of these tissues give rise to the very high $\chi_{\nu }^{2}$.

**TABLE 2:** Reduced Chi‐square of the result of the fitted minimizer for each mouse, both for the short scale and for the long scale.

	Reduced Chi‐square	
Mouse	Short scale	Long scale
16A0818A	1.05	25.03
16A0818B	0.84	23.01
16A0823	2.57	249.13
17A1012C	1.78	44.99
17A1101A	6.94	194.58
17A1101B	3.15	46.42

**TABLE 3:** Mean contribution to the reduced Chi‐square of the result of the fitted minimizer of each organ, both for the short scale and for the long scale.

	Reduced Chi‐square	
Organ	Short scale	Long scale
Heart	0.95	7.96
Lungs	0.66	42.58
Liver	0.56	13.71
Kidneys	0.19	23.48
Femur	0.32	8.74

### 3.2 Parameter estimates

**FIGURE 4:**
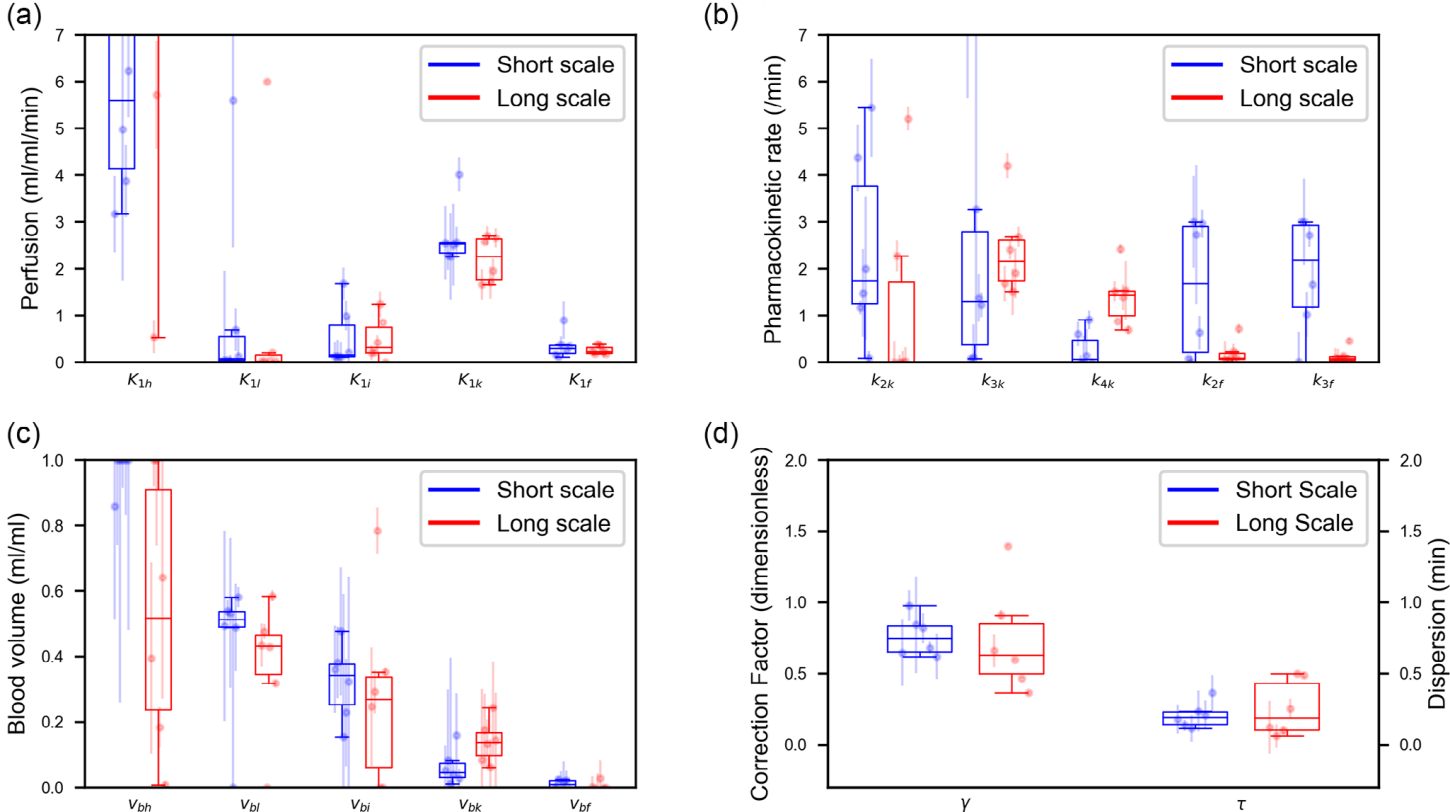
Boxplots of the parameter estimations of the six fits, including the estimates for the perfusion values (a), other pharmacokinetic parameters (B), the blood volume (c), and the correction coefficients of the arterial input function (d). The single estimates for mice 1 to 6 in that order are plotted alongside each box.

Values derived from the two analysis approaches can be compared starting from the box plot in Figure 4. For *K_1_* (Figure 4a), the heart does not have consistent estimates of uptake rate either on the short scale (first 3 min) or the long scale (60 min). For the other organs, estimates are both fairly consistent and precise, with low standard deviations. The uptake rate in the lungs is very small, especially when fitted to the long scale data, being *K_1 _* = 0.06 ± 0.08 mL/mL/min when discarding one outlier of *K_1 _* = 6 mL/mL/min. The uptake rate to the liver is larger and the estimates are less consistent, with *K_1 _* = 0.49 ± 0.43 mL/mL/min for the long scale data. For the heart, lungs, and liver, the value of *k_2_* is determined by dividing *K_1_* by the volume of distribution. The estimates of *K_1_* for the kidneys and femur are very consistent, especially when considering the full hour of data, where *K_1 _* = 2.21 ± 0.45 mL/mL/min and *K_1 _* = 0.26 ± 0.08 mL/mL/min, respectively. The uptake rate in the kidneys is particularly high, which is expected. For the remainder of the kidney parameters (Figure 4b), reliability is a problem, for example, on a short timescale *k_2_* = 2.43 ± 2.05/min. The *k_3_* is only estimated consistently when considering the longer timescale, where *k_3_* = 2.40 ± 0.90/min. *k_4_* has two different but consistent estimates for the two timescales (0.27 ± 0.35 and 1.40 ± 0.56/min for the short and long scales, respectively), where the estimate becomes higher as longer timepoints are considered.

For the femur (Figure 4b), *K_1_* can be estimated for both scales, the parameters *k_2_* and *k_3_* cannot be separated unless the full hour of data is considered, where *k_2_* = 0.20 ± 0.24/min and *k_3_* = 0.14 ± 0.15 /min. A combination of both parameters as well as *K_1_*, the influx rate of tracer *K_i_* = *K_1_* ×*k_3_*/(*k_2_* + *k_3_*) (Table 1) can, however, be estimated at any scale. The estimates for the blood volume of the liver, kidneys and femur are especially robust (Figure 4c). The estimates for the heart and lungs are less consistent, especially when considering the long scale data. In the case of the short timescale fit, the estimates for the blood volume in the heart are almost all *v_bh _* = 1, but with high uncertainty (Table 1).

The corrections to the AIF measured by the PET scanner are positive for both timescales (Figure 4d). The correction factor γ and dispersion τ estimates are more varied when the model is fitted to the full hour of data, but remain within a reasonable range. Delays for all tissues are successfully estimated for both timescales and are consistent and small (Table 1).

### 3.3 Multi‐parameter dependencies for different tissues

**FIGURE 5:**
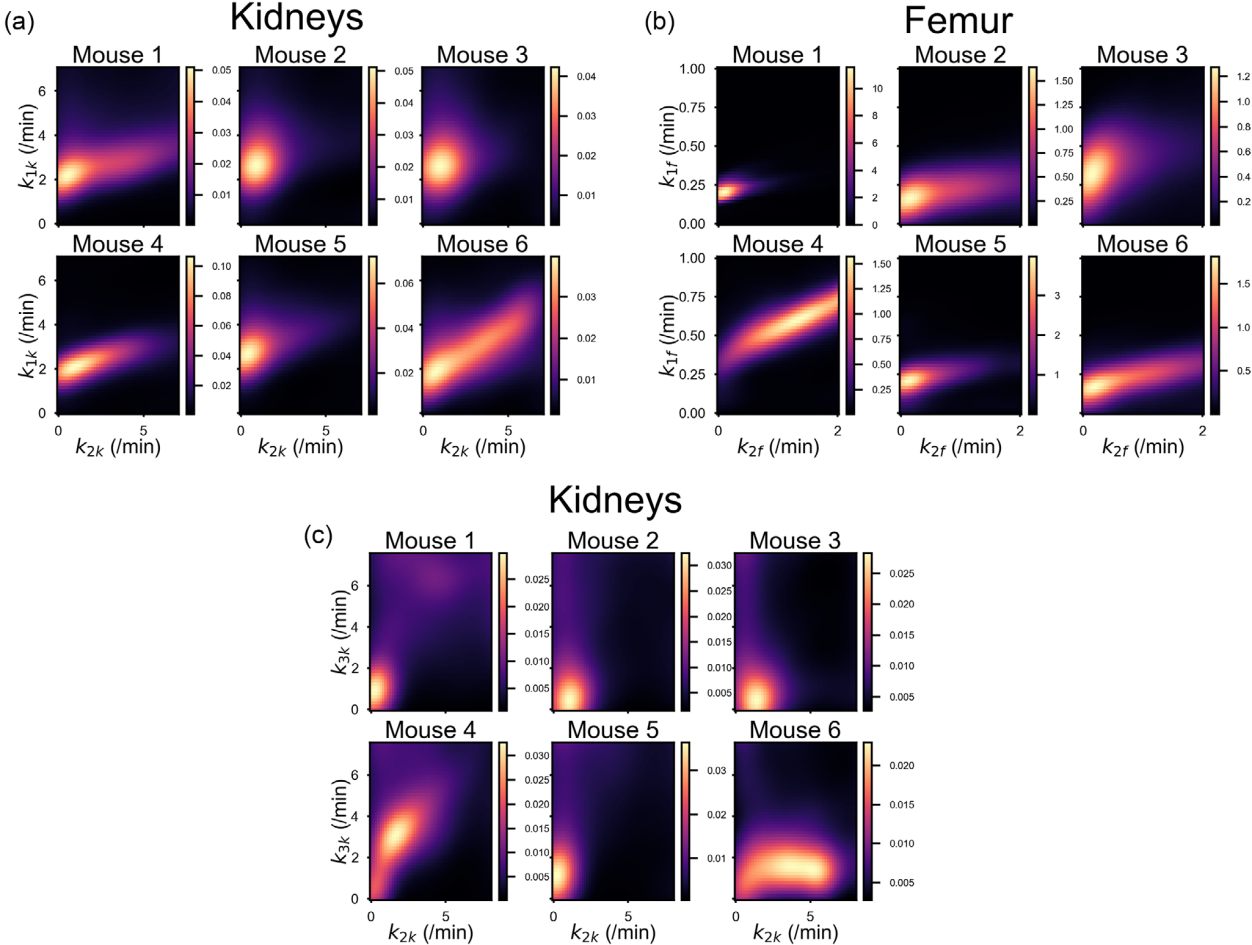
Kernel density estimates of the distribution of results in two‐dimensional parameter space, showcasing the relationships between K_1_ and k_2_ in the kidneys (a), K_1_ and k_2_ in the femurs (b), and k_2_ and k_3_ in the kidneys (c).

**FIGURE 6:**
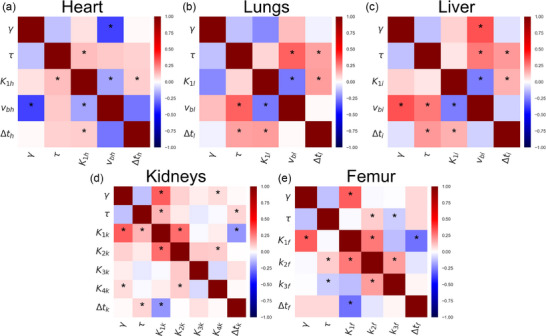
Individual correlation heatmaps in the short scale of the heart (a), lungs (b), liver (c), kidneys (d), and femurs (e). The * symbols indicate significant correlations as described in Section 2.8.

**FIGURE 7:**
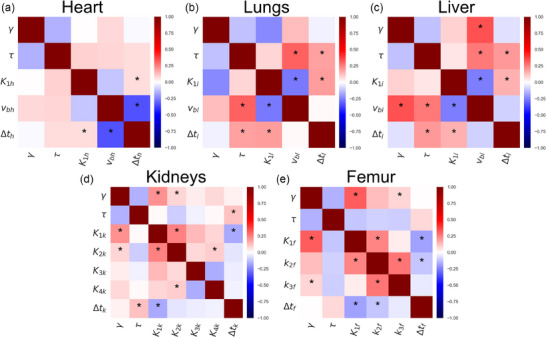
Individual correlation heatmaps in the long scale of the heart (a), lungs (b), liver (c), kidneys (d), and femurs (e). The * symbols indicate significant correlations as described in Section 2.8.

Success determining parameters for all organs and both timescales is summarized qualitatively in Table 4, and all correlations are summarized in Figures S1 and S2. This phenomenon is presented as the probability distribution of the estimated microparameters, Figure 5, for the cases of *K_1_* and *k_2_* for both the kidneys and the femur (and *k_2_* and *k_3_* for the kidneys) and via correlation heatmaps for the remaining organs (Figures 6 and 7).

**TABLE 4:** Summary of the ability of the minimizer to estimate parameters, for the short scale (3 min) and long scale (60 min).

	γ	τ	Δt_h_	Δt_l_	Δt_i_	Δt_k_	Δt_f_	v_bh_	v_bl_	v_bi_	v_bk_	v_bf_
Short scale	Yes	Yes	Yes	Yes	Yes	Yes	Yes	Yes	Yes	Yes	Yes	Yes
Long scale	Yes	Yes	Yes	Yes	Yes	Yes	Yes	No	No	Yes	Yes	Yes

	K_1h_	K_1l_	K_1i_	K_1k_	k_2k_	k_3k_	k_4k_	K_1f_	k_2f_	k_3f_
Short scale	Yes	No	Yes	Yes	No^a^	No	No	Yes	No^a^	No
Long scale	No	No	Yes	Yes	No^a^	Yes	Yes	Yes	Yes	Yes

^a^ Parameter cannot be estimated due to clear correlations with other parameters.

## 4 DISCUSSION

Pharmacokinetic modeling of PET data is a routine research approach for quantitatively characterizing blood flow, calcification, metabolic rate, or receptor density. Typically, a single model is used appropriate to the choice of tracer and the tissue of interest. The advent of total‐body PET scanners will make it possible to quantitatively characterize many different organs simultaneously permitting a whole systems approach. This study aimed to explore multi‐organ pharmacokinetic modelling for the PET tracer Na[^18^F]F using murine datasets and focusing on five organs. Multi‐organ pharmacokinetic modeling has previously been applied in the context of simultaneous estimation (SIME) of an input function.^42^ That approach is quite different to the work presented here because it does not involve an independent determination of the input function. Our results show that kinetic analysis can be carried out for multiple organs in a single step allowing flow to be characterized for different organs; however, correlations between parameters present a marked challenge.

Existing total‐body PET studies have used the tracer 2‐[^18^F]‐fluoro‐2‐deoxy‐d‐glucose (^18^F‐FDG)^21^, ^22^, ^23^, ^24^, ^43^ and, where compartmental models have been used, a single model has been assumed.^21^, ^22^, ^23^, ^24^ The typical focus of these studies was to quickly extract the influx rate, *K_i_*, or the uptake rate, *K_1_*, in order to reduce scan time while still accurately pin‐pointing lesions. Capturing the full behavior of very different tissues with widely ranging response times has not previously been considered. In the present study, two different timescales for kinetic modeling were compared for multiple organs because ultimately, reducing the scan duration would expedite the clinical use of Total‐body PET.

The choice of compartmental model for each tissue was informed both on the expected pharmacokinetic behavior of Na[^18^F]F and time‐activity curves observable kinetics. Unlike in the case of ^18^F‐FDG, a wider variety of models is required.^21^, ^22^, ^23^, ^24^ The models give a good qualitative account of the activity curves (Figure 3, parameters Table 1) irrespective of the duration of the data analyzed. If a single analysis approach is to be taken with all organs, then, based on $\chi_{\nu }^{2}$ values, the analysis of the first 3 min of data is preferable to analysis of the full hour. The models nonetheless fit the important aspects of the tissue activity curves over the full hour, and some divergence from reality is expected due to the simplicity of the models. The exception was the femur, for which it was advantageous to model the full hour of data. We note that focused investigations of bone metabolism using Na[^18^F]F can record PET data for up to 120 min, consistent with this finding.^44^ For the other organs, more elaborate models could have been chosen to more completely describe the full hour of data; however, this would have led to a proliferation of free parameters and hence larger uncertainties in the estimates. When the model is fitted to the short timescale data (Figure 3a), the average $\chi_{\nu }^{2}$ among all fits is 2.72, close to the desired value of 1 (Table 2). The major contributor to this error is the heart activity, with 35% of the total error, and the lungs, with 25% (Table 3). The kidneys (7%) and femur (12%) curves contribute the least to the $\chi_{\nu }^{2}$. In the model for the kidneys used here, the radiotracer is removed from the second compartment through a clearance to the bladder rate *k_4_*. For both the femur and the kidneys, no restriction is imposed on the relationship between *K_1_* and *k_2_*. In spite of the low $\chi_{\nu }^{2}$, estimating the value of some parameters can be a problem. For example, difficulties extracting values (*k_3_*) and consistent values (*k_4_*) for the kidneys using the short time‐scale data perhaps reflect the slow action of the kidneys when filtering the radiotracer.

There are specific challenges accurately modeling the data from the lungs and the liver toward the end of the 60 min of data. The activity varies rapidly in the lungs, and at the temporal resolution of these measurements, there is little difference between these data and the IDIF. This places a strain on the modeling process. Temporal resolution is not such a challenge for the liver. As described above, in order to avoid a proliferation of parameters, our model of the liver has been kept simple. It is well known that the liver has a dual blood supply and that the tracer arriving through the portal vein tends to be highly dispersed. This effect is not included in our model, and this is likely to cause problems with obtaining a perfect correspondence with the complete data set.

The extraction fraction for Na[^18^F]F has only been thoroughly studied for the case of vertebra in a mini pig model.^44^ Although this will not translate directly to other tissues and other species, it can reasonably be assumed that the extraction fraction will only be close to 1 for low flow values (say around 0.1 mL/mL/min). On this basis, *K_1_* can be compared to the anticipated flow for the lungs, liver, and femur (Figure 4). Using published volume flow rates^45^ and tissue densities^46^ for mice, it is anticipated that *F*(lungs) = 0.107 mL/mL/min and *F*(liver) = 0.177 mL/mL/min. Equivalent values for the femur for mice are not available. We found *K_1_* (lungs, short) = 0.184 ± 0.413 mL/mL/min; *K_1_* (lungs, long) = 0.058 ± 0.003 mL/mL/min; and *K_1_* (liver, short) = 0.540 ± 0.177 mL/mL/min and *K_1_* (liver, long) = 0.492 ± 0.058 mL/mL/min. In the case of the lungs, one outlier was removed. Evidently, the values for the liver are noticeably above expectations (although four mice had values close to expectation), whereas the lungs reveal values roughly in line with expectations.

The flow is expected to be larger for the kidneys, meaning that the extraction fraction can no longer be assumed to be close to 1. Hence, for both the kidneys and the femur, we are not able to make a comparison with literature values for flow. It is interesting to note that for each of these organs, the sets of *K_1_* values determined for different timescales are consistent with each other. that is, the determination of *K_1_* is independent of whether 3 or 60 min of the data is used for analysis.

The challenge of establishing the values of microparameters is partly because their values cannot be independently optimized due to correlations. In the extreme case, this can result in a failure to estimate the value of a microparameter. There are some difficulties for all organs (see Supplementary Information), here we focus on the kidneys and the femur. The large number of parameters to fit for the femur and the kidneys makes finding the optimal fit challenging due to correlations between parameters. One part of this is the correlations between *K_1_* and *k_2_*, which is a problem that has been noted before in the context of analyzing organs separately.^47^, ^48^ For the kidneys, the pharmacokinetic parameter *k_2_* in the short scale could not be separated. However, an almost linear relationship between *K_1_* and *k_2_* is observed for half of the subjects (Figure 5a). For those animals, an analogue of the volume of distribution for the one‐compartmental tissues could be estimated. This relationship between the two parameters is however less important when fitting the entire hour of data (Figure 7d). On the other hand, *k_3_* in the short timescale fits presented weaker correlations (not reaching significance) with the uptake rate to the kidneys and their clearance to the bladder rate (Figure 5c), and the specification of two of these three parameters might help reduce the uncertainty of the third parameter considerably. The situation with *K_1_* and *k_2_* for the femur is slightly better (Figure 5b). For four of the subjects, these microparameters are centered on an optimal value rather than exhibiting proportionality. When considering the femur, *k_2_* and *k_3_* cannot be separated in the short scale. Indeed, the femur is the organ where the timescale matters most (Figure 4b). These challenges with correlations between parameters strongly indicate that there is a limit to how flexible our model can be and hence how many parameters we can attempt to estimate. Further discussion of Figures 6 and 7 can be found in the Supplementary Information.

The problem of correlations between parameters could be mitigated by reducing the number of parameters. This might be achieved by, for example, fixing the value of the blood volume in each organ. This would clearly loose the sensitivity to the details of the specific subject, however, typical values of the blood volume for each organ are known which would help at least. The remaining parameters would immediately become more stable. Other improvements would be more challenging. As described above, in order to improve the modeling of the heart and lungs, it would be essential to reconstruct PET images at higher temporal resolution. While this might be very difficult in the pre‐clinical context, this would be much more straightforward for clinical scans using total body PET. In order to improve the modeling of other organs, a delicate balance between improvements and increased numbers of parameters would need to be struck. As mentioned, the liver should really have a dual blood supply, but it is not obvious that the additional parameters will improve our multi‐organ model overall.

This work shows that multi‐organ models with a shared cardiovascular system can be used to model preclinical PET data for mice using the tracer Na[^18^F]F. This is a prototype example of modeling a biological system. Our study suggests that such an analysis would perform best if the model fitting was carried out for different time periods for different organs. Most organs would benefit from model fitting for 3 min of data, whereas the femur benefits from model fitting for the full hour of data. Our analysis of the correlations between parameters contributes to our understanding by demonstrating that the number of parameters in the model must not become too large. With our model, it has been possible to extract perfusion values in some cases. Potentially, the understanding gained from this preclinical study could be taken on to clinical total body PET studies using Na[^18^F]F. The aim would be to quantify both micro‐calcification and perfusion across multiple organs and tissues in the whole‐body. Due to the higher temporal and spatial resolution of clinical total body PET scanners, some of the challenges with modeling the heart and the lungs, for example, would be much less problematic.

A limitation of the current work is the requirement for general anesthesia during the imaging of the mice. It is well‐known that isoflurane can have an influence on tissue perfusion.^49^ Our quantitative PET results should be considered in this light. Nonetheless, the use of general anesthesia does not undermine our broader conclusions about the value of modeling a whole system. Our model couples all organs together via a shared cardiovascular system. This situation has the potential to induce cross‐talk between separate organs. For example, if the optimal estimates of the correction factor γ and dispersion τ were similar for most organs but quite different for the femur, this would be likely to have a negative influence on the estimates of other microparameters for the femur. Likewise, via the shared cardiovascular system, parameters from one organ could become correlated with those of a different organ. In practice, it appears that this is not an important effect (see Supplementary Information, Figures S1 andS2). The strong correlations are found close to the leading diagonal, indicating parameters within the same organ, or are associated with the corrections to the IDIF. Consideration of parameter correlations between organs and how they vary with model choice would be worthy of further attention in the future.

## 5 CONCLUSION

We have demonstrated an approach to performing kinetic modeling for multiple organs and a shared cardiovascular system simultaneously for the example of the PET tracer Na[^18^F]F. This is challenging particularly due to correlations between microparameter values, a problem which must be overcome in order to perform systems‐level analysis of the new total‐body PET data. An appropriate model and scan duration is required for each organ, for example, the femur benefits from a longer scan, whereas the other organs are better analyzed over less time. Correlations between microparameter values were demonstrated to degrade analysis for the kidneys and the femur. However, for other organs, microparameters can be estimated reliably with a quantitative analysis of perfusion being possible for some organs. When conducting multi‐organ total‐body PET analysis, degree of dependencies of different kinetic constants should be quantified and weighted alongside goodness of fit and parameter estimates quality.

## CONFLICT OF INTEREST STATEMENT

The authors declare no conflicts of interest.

## Supporting information



Supporting Information

## Data Availability

The data that support the findings of this study are available upon reasonable request from the authors.
