# Supplementary material for: Multi‐organ kinetic modeling for Na[18F]F pre‐clinical total‐body PET studies
Source: Med Phys. 2024 Nov 5;52(2):924–37. doi: 10.1002/mp.17499 (PMC11788250; doi:10.1002/mp.17499)
Supplement: Supplementary file 1 — Supporting Information [file MP-52-924-s001.pdf]

Supplementary Information: Multi-organ kinetic modelling for Na[^{18}F]F pre-clinical total-body PET studies

By: Jose Benitez-Aurioles[1, 2], Paul S. Clegg[1], Carlos J. Alcaide-Corral[3,5], Catriona Wimberley[1], and Adriana A. S. Tavares[3] (Email. adriana.tavares@ed.ac.uk)

Affiliations:

[1]School of Physics & Astronomy, University of Edinburgh, Edinburgh, EH9 3FD, UK

[2]University of Manchester, Division of Informatics, Imaging and Data Science, Manchester, M13 9PL, UK

[3]Edinburgh Imaging, University of Edinburgh, Edinburgh, EH16 4SB, UK

[4]Centre for Clinical Brain Sciences, University of Edinburgh, Edinburgh, EH16 4SB, UK

[5]University/BHF Centre for Cardiovascular Science, University of Edinburgh, Edinburgh, EH16 4TJ, UK

Parameter correlations beyond the kidneys and femur:

The correlation between parameters for the kidneys and femur have been presented in Figures 5, 6 and 7 and considered in the Discussion section. In other organs problems were also observed, often associated with the blood volume. For the lungs on the short scale, the uptake rate shows a strong inverse correlation with the blood volume (Figure 6B). A pronounced correlation is also observed between the blood volume in the lungs and the correction coefficient in the fits to the long timescale data (Figure 7B). For the liver on the short scale, there were difficulties in estimating the liver uptake rate consistently. This can be partially explained by high correlations between this parameter and the correction coefficient and the blood volume of the tissue (Figure 6C). Similarly, the blood volume of the heart is inversely correlated with the arterial input function coefficient on the long scale, at least for half of the

animals (Figure 6A). More fundamentally, the high blood volume of the heart means that the contribution of the perfusion to microparameter K_1 is minimal and hence cannot be estimated.

Correlation heatmaps for all parameters across all organs are presented in Figures S1 and S2. Here it can be seen that the most pronounced correlations occur either close to the leading diagonal or are associated with the corrections to the image derived input function. These observations indicate that correlations are primarily for parameters within a single organ. Strong correlations between parameters from different organs are not observed.

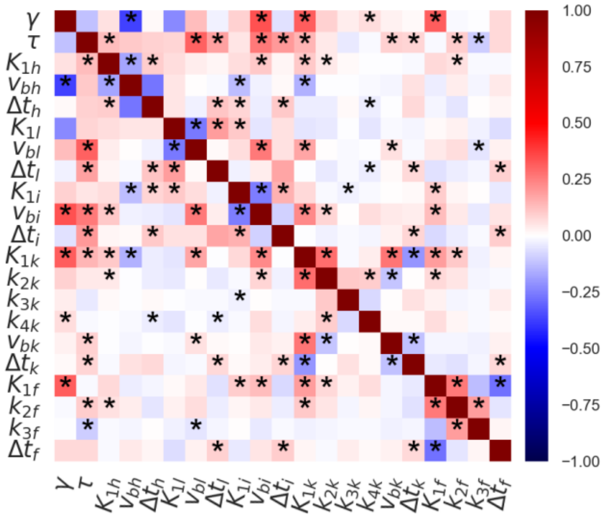

Fig. S-1. Correlation heatmaps for all organs simultaneously for the short timescale. The * symbols indicate significant correlations as described in Section 2.8.

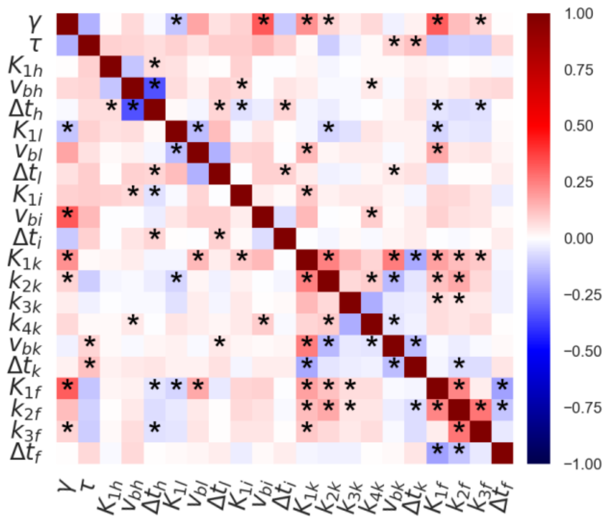

Fig. S-2. Correlation heatmaps for all organs simultaneously for the long timescale. The * symbols indicate significant correlations as described in Section 2.8.
